# Fine-Tuned Temporal Dense Sampling with 1D Convolutional Neural Network for Human Action Recognition

**DOI:** 10.3390/s23115276

**Published:** 2023-06-02

**Authors:** Kian Ming Lim, Chin Poo Lee, Kok Seang Tan, Ali Alqahtani, Mohammed Ali

**Affiliations:** 1 Faculty of Information Science and Technology, Multimedia University, Melaka 75450, Malaysia; 2 Department of Computer Science, King Khalid University, Abha 61421, Saudi Arabia; 3 Center for Artificial Intelligence (CAI), King Khalid University, Abha 61421, Saudi Arabia

**Keywords:** human action recognition, temporal dense sampling, 1D convolutional neural network (1D ConvNet), 1D-CNN, Inception-ResNet-V2

## Abstract

Human action recognition is a constantly evolving field that is driven by numerous applications. In recent years, significant progress has been made in this area due to the development of advanced representation learning techniques. Despite this progress, human action recognition still poses significant challenges, particularly due to the unpredictable variations in the visual appearance of an image sequence. To address these challenges, we propose the fine-tuned temporal dense sampling with 1D convolutional neural network (FTDS-1DConvNet). Our method involves the use of temporal segmentation and temporal dense sampling, which help to capture the most important features of a human action video. First, the human action video is partitioned into segments through temporal segmentation. Each segment is then processed through a fine-tuned Inception-ResNet-V2 model, where max pooling is performed along the temporal axis to encode the most significant features as a fixed-length representation. This representation is then fed into a 1DConvNet for further representation learning and classification. The experiments on UCF101 and HMDB51 demonstrate that the proposed FTDS-1DConvNet outperforms the state-of-the-art methods, with a classification accuracy of 88.43% on the UCF101 dataset and 56.23% on the HMDB51 dataset.

## 1. Introduction

Human action recognition is a crucial area of research in computer vision, with applications in areas such as pedestrian detection and video surveillance systems. It involves classifying a human action video into its relevant activity category and is a challenging task due to the spatiotemporal nature of videos. These challenges include, but are not limited to, occlusion, similar actions, and the varying durations of human actions.

Human action recognition can be divided into two main categories: hand-crafted methods and representation learning. Hand-crafted methods involve extracting low-level features such as edges and corners from both spatial and temporal dimensions of the video, and combining them to represent video-level features. This approach is computationally complex and less favored for real-time applications. Representation learning, on the other hand, involves training and tuning a model to automatically discover discriminative features. One of the most popular methods in representation learning is convolutional neural networks (ConvNets). However, conventional ConvNets designed for 2D image recognition may not fully utilize the temporal information of the human action video.

In this paper, we propose the fine-tuned temporal dense sampling with 1D convolutional neural network (FTDS-1DConvNet) for human action recognition. A fine-tuned temporal dense sampling (FTDS) strategy is first utilized to partition the human action video into segments and passes each segment through a fine-tuned Inception-ResNet-V2. Temporal max pooling is applied to all frames in the segment along the temporal axis to perform long-range subsampling. Subsequently, the output of the FTDS strategy is then fed into a 1D convolutional neural network (1DConvNet) for representation learning and classification. This dense sampling method subsamples the frames of the video to better capture the spatial and temporal information, and converts human action videos of varying lengths into a fixed-length spatiotemporal representation.

The main contributions of this paper are:

1. A fine-tuned temporal dense sampling strategy that captures the significant spatial information and long-term dynamics of the human action video in a fixed-length representation.
2. A single-stream 1DConvNet with FTDS that effectively represents the spatial information and temporal information of the human action video. In addition, FTDS-1DConvNet also reduces the possible temporal information loss of single-stream RGB-based ConvNet methods and the high computational requirements of LSTM methods.

## 2. Related Work

Over the years, techniques and optimizations in image and video processing have been fundamentally similar. Generally, the procedures in image and video processing are separated into: preprocessing, feature extraction, and classification. The videos are typically stored in a 3D format, which is also interpreted as the spatial changes over the time. Since the early stages of research in human action recognition, most related works have been devoted to the understanding of these spatiotemporal changes. Generally, there are two main categories in human action recognition: hand-crafted feature and representation learning. In the computer vision community, hand-crafted based methods were originally a concept to quantize the representative properties of an image. The hand-crafted based methods manually design an algorithm to discover the data representations, which is also known as feature engineering [1]. The obtained features are then fed into a classifier to perform classification for a specific domain. Therefore, the quality of the features heavily affects their performance, which was also the main scope for most of the early work.

### 2.1. Hand-Crafted Methods

Early work on hand-crafted methods in human action recognition focused on the development of temporal templates. Bobick and Davis introduced the motion energy image (MEI) and motion history image (MHI) [2] to represent the motion information in a video. The MEI represented motion as a binary template while the MHI captured the motion history over time. A later work, by Kim et al., introduced the accumulated motion image (AMI) [3], which computed the average of differences between consecutive frames, but it was found that its performance was limited by variations in appearance. Blank et al. [4] represented human actions as a series of silhouettes over time, referred to as space-time shapes. They used the Poisson equation [5] to extract meaningful spatiotemporal features from the space-time shapes. Another work, by Yilmaz et al. [6], characterized human actions by computing correspondences between contours in consecutive frames, resulting in the spatiotemporal volume (STV) representation.

Later, Laptev et al. [7] proposed the use of histograms of optical flow (HOF) to represent motion direction and magnitude. They combined HOF with histograms of oriented gradient (HOG) [8] and applied the bag of visual words (BOV) representation. However, the lack of realistic and annotated datasets under uncontrolled environments presented a challenge. To address this, they collected a real-world dataset from Hollywood movie clips in addition to the KTH dataset and proposed an automated annotation algorithm using information from movie scripts. Klaeser et al. [9] computed a 3D version of HOG, known as HOG3D, to address the issue of descriptor variability under different lighting conditions.

Oneata et al. [10] combined motion boundary histogram descriptors with scale-invariant feature transform (SIFT) to form a Fisher vector (FV) representation. A simplified version, the vector of locally aggregated descriptors (VLAD) [11], was introduced to balance between BOV and FV. Peng et al. [12] conducted experiments with various combinations of local descriptors and aggregation encoders, including BOV, FV, and VLAD. They concluded that a hybrid supervector representation based on the improved dense trajectory was a good representation for human action recognition. Despite the success of hand-crafted methods, the design of features or descriptors often requires extensive manual effort. To address this limitation, recent research has explored representation learning to reduce the need for manual design.

### 2.2. Representation Learning Methods

Representation learning, also known as feature learning, is a subfield of machine learning that focuses on discovering the underlying representations of data in an end-to-end manner [1]. In the context of human action recognition, recurrent neural networks (RNN) and convolutional neural networks (ConvNet) are the most commonly used neural-network-based architectures. The use of neural networks in this field is not only due to their ability to model the data representations, but also because they can be incorporated with classifiers, making them highly flexible and adaptable [13]. One of the challenges in training RNNs is the vanishing and exploding gradients problem during backpropagation, as pointed out by Bengio et al. [14]. To address this, Hochreiter and Schmidhuber introduced long short-term memory (LSTM) networks, which have additional memory cells in their hidden layers to prevent the vanishing gradient problem [15]. As a result, LSTM networks have become the preferred choice over traditional RNNs in human action recognition.

Donahue et al. [16] integrated an LSTM network into a two-stream ConvNet to preserve spatiotemporal information from both RGB and optical flow streams, while Ng et al. [17] extended this with an LSTM network composed of five hidden layers. However, Shi et al. [18] argued that the two-stream attentional LSTM network could result in the optical flow stream dominating over the RGB stream during training. To overcome this, they limited the information exchange between the two streams and only shared certain layers during training. Additionally, they adopted a gated recurrent unit (GRU) network [19] instead of a traditional LSTM network. Wang et al. [20] proposed the temporal segment network (TSN) to learn human actions by dividing videos into segments and feeding them into a two-stream ConvNet. Ma et al. [21] further improved TSN by feeding multiple segments sequentially into an LSTM network, where each segment was represented by the concatenation of features from both the spatial and temporal networks. Pan et al. [22] introduced the cross-stream selective network (CSN), which used a bidirectional long short-term memory (BiLSTM) network to select the most correlated optical flow stack for each RGB segment and compute attention scores for each segment in the CSN. In a previous work [23], BiLSTM with temporal dense sampling for human action recognition was proposed. The temporal dense sampling divided the video of human action into sections and then conducted a max pooling operation along the temporal axis within each of those sections. This technique captured a dense set of temporal features, which can improve the model’s ability to recognize complex actions. To better capture the long-term spatial and temporal dependencies present in the data, the multi-stream BiLSTM network was proposed. The network encoded information in both the forward and backward directions, allowing it to capture temporal dependencies in both the past and future frames. Moreover, by leveraging bidirectional information, the model can better understand the context of the action being performed.

While the majority of RNN-based human action recognition approaches are two-stream architectures, there is a limited number of works addressing the issue of long and varying temporal structures in human action videos. Initially, ConvNet was designed for 2D images, but it was later extended to handle human action videos by extending convolution and pooling along the time axis [24]. However, ConvNets typically require large datasets, and the size of the existing datasets in human action recognition was insufficient. To overcome this, Ji et al. [24] extracted hand-crafted features from human action sequences and fed them into a 3D ConvNet, while Karpathy et al. [25] collected a larger dataset called Sports-1M, with 1 million human action videos, to train a 2D ConvNet for human action recognition. They proposed a 2D ConvNet with a two-stream architecture, i.e., context stream and fovea stream. In the context stream, they down-sampled the original resolution by half. As for the fovea stream, they centrally cropped the selected frames before the down-sampling operation.

Spatial-temporal features have been extensively explored in the field of human action recognition, with a variety of methods and architectures proposed. Yan et al. [26] proposed a novel deep learning architecture for action recognition from skeleton data. The proposed architecture, called spatial temporal graph convolutional networks (ST-GCN), was based on graph convolutional networks (GCNs) and was designed to process the skeletal joint data obtained from motion capture sensors. The ST-GCN architecture utilized the structural information inherent in the skeleton data by modeling it as a graph, where the joints were represented as nodes and the edges represented the spatial and temporal relations between the joints. Later, Zhu et al. [27] integrated spatial and temporal knowledge under challenging conditions such as occlusions, pose variations, and lighting changes. This approach used a two-stage pipeline consisting of a feature extraction module and a landmark tracking module. Another work, by He et al. [28], utilized both local and global spatial-temporal information to model the spatiotemporal dynamics of human actions, named as STNet. The STNet architecture was composed of two main components: a local spatial-temporal modeling module and a global spatial-temporal modeling module. They used 3D CNNs and temporal attention mechanisms to capture both short-term and long-term temporal dynamics. Yao et al. [29] proposed a novel deep learning architecture called the spatial-temporal dynamic network (STDN), which used a flow gating mechanism to learn the dynamic similarity between locations. Additionally, a periodically shifted attention mechanism was utilized to handle long-term periodic temporal shifting in the input data.

In recent years, several variations of the two-stream ConvNet architecture have been proposed by researchers for human action recognition. Wang et al. [30] presented a deep two-stream ConvNet using VGGNet-16 [31] and GoogleNet [32] and further improved it with various training techniques. Yifan et al. [33] argued that a full understanding of visual elements is crucial for human action recognition and, therefore, used a pre-trained Faster R-CNN [34] in a two-stream ConvNet architecture to model the representations of three visual semantics (background, human, and object). This was achieved by fusing meaningful information from both streams using four fusion strategies: sum, max pooling, category-wise weighted, and correlation-wise weighted. Christoph and Pinz [35] introduced Spatiotemporal-ResNet (ST-ResNet) to learn the interaction between both streams by injecting residual connections from the motion stream into the spatial stream, taking advantage of the impressive performance of ResNet [36].

However, computational limitations have led to the neglect of long-term temporal structure in human action videos in two-stream ConvNet-based architectures. To address this issue, Varol et al. [37] introduced a successful attempt at long-term modeling by examining the effect of different frame sampling rates (12, 20, 40, 60, 80, 100) in their two-stream 3D ConvNet. Wang et al. [20] proposed a temporal segment network (TSN) to learn human action videos by dividing them into segments, selecting one frame and a stack of optical flow in each segment, and feeding it into a two-stream ConvNet. This method showed promising results on the UCF101 and HMDB51 datasets, even without transfer learning from a large-scale human action dataset. However, it is questionable whether a complete human action video can be expressed with only one frame in each segment.

Despite the impressive results obtained by 3D ConvNet, they are more complex than 2D ConvNet and require more trainable parameters, which makes them heavily reliant on large-scale datasets and computationally prohibitive. To overcome these limitations, we investigate the potential of integrating the RGB stream and temporal dense sampling into 1D ConvNet.

## 3. Fine-Tuned Temporal Dense Sampling with 1D Convolutional Neural Network (FTDS-1DConvNet)

In this work, we introduce a novel method for human action recognition, fine-tuned temporal dense sampling with 1D convolutional neural network (FTDS-1DConvNet). The method consists of two components: fine-tuned temporal dense sampling (FTDS) and 1D ConvNet. The FTDS component partitions the human action video into a fixed number of segments, and then fine-tunes a pre-trained Inception-ResNet-V2 on the human action dataset. The fine-tuned Inception-ResNet-V2 serves as the spatial encoder of the segment, converting human action videos of varying lengths into fixed-length representations through a max pooling operation along the temporal axis.

**Figure 1:**
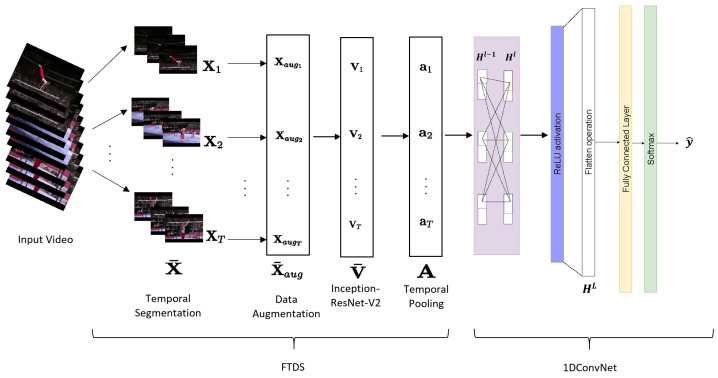
The proposed FTDS-1DConvNet for human action recognition.

The output of the FTDS component is then passed into the 1D ConvNet component for representation learning and classification. The 1D ConvNet has fewer trainable parameters than 2D ConvNet and LSTM, making it more computationally efficient. The convolution layer, pooling layer, and fully connected layer in the 1D ConvNet serve as feature extractors, learning high-level representations for the input. The classification layer computes the probability distributions for the human action class using the softmax function. The final class label is assigned to the human action with the highest probability distribution. The architecture of the FTDS-1DConvNet is illustrated in Figure 1.

### 3.1. Preprocessing with Temporal Segmentation and Data Augmentation

The proposed preprocessing method consists of two stages: temporal segmentation and data augmentation. The first stage of preprocessing, temporal segmentation, involves dividing the human action video into a fixed number of segments. This enables the transformation of human action videos of varying lengths into fixed-length representations.

Given a human action video with a total of *N* frames, the video is equally divided into *T* segments to generate:

(1) $$\bar{X}=\left\{X_{1},X_{2},\ldots ,X_{T}\right\},\;X_{t}\in R^{(h\times w\times c)\times n},\;t=1,2,\ldots ,T$$

where each segment is composed of $n=\frac{N}{T}$ frames. The height, width, and channels of each frame are represented by *h*, *w*, and *c*, respectively. By collecting all $X_{t}$ over *T* segments, a set of matrices $\bar{X}$ is formed, and fed into data augmentation for further preprocessing.

The second stage of preprocessing, data augmentation, is applied to the segments generated from the temporal segmentation. The objective of data augmentation is to increase the number of training samples and to reduce overfitting in the model. Two data augmentation techniques, random cropping and random horizontal flipping, are applied to $\bar{X}$. In random cropping, a sub-region with the ratio of 0.875 is randomly cropped from each frame. The cropped sub-region is then randomly flipped horizontally. It is important to note that the data augmentation is applied at the video level, ensuring that every frame is consistently cropped and flipped throughout the entire video. Thereafter, the height and width of these augmented frames are rescaled to 299×299 for use as the input of the Inception-ResNet-V2. Finally, the pixel intensity is normalized to a range of [−1, 1] to stabilize the training:

(2) $${\bar{X}}_{aug}=\left\{X_{aug_{1}},X_{aug_{2}},\ldots ,X_{aug_{T}}\right\},\;X_{aug_{t}}\in R^{(h\times w\times c)\times n},\;t=1,2,\ldots ,T$$

where ${\bar{X}}_{aug}$ is a set of augmented input streamed to the Inception-ResNet-V2.

### 3.2. Transfer Learning with Inception-ResNet-V2

In this work, the pre-trained Inception-ResNet-V2 model on ImageNet [38] is leveraged as the initial weights for fine-tuning on human action videos. The augmented input set, ${\bar{X}}_{aug}$, is fed into the Inception-ResNet-V2 model to extract a set of features, represented as:

(3) $$\bar{𝒱}={𝒱}_{1},{𝒱}_{2},\ldots ,{𝒱}_{T},\;{𝒱}_{t}\in R^{d\times n},\;t=1,2,\ldots ,T$$

where *d* is the dimensionality of the output from a selected layer and *T* represents the number of frames. To preserve the prior knowledge gained from the ImageNet dataset, a global average pooling layer is employed. Each frame is transformed into a feature vector with 1536 dimensions, resulting in a feature matrix ${𝒱}_{t}$ of dimensions 1536×n. After transfer learning with Inception-ResNet-V2, temporal pooling is used to account for all frames in a segment, leading to dense sampling along the temporal axis using the proposed FTDS.

The feature matrix ${𝒱}_{t}$ obtained from the fine-tuned Inception-ResNet-V2 is a comprehensive representation of *n* frames at time step *t*. However, due to its large size and varying number of frames, it is not feasible to input this matrix directly into the recognition network. The conventional solution is to use sparse sampling, where a single frame is randomly selected from each segment. This approach can lead to significant information loss. To overcome this challenge, a new subsampling technique called temporal pooling is introduced. Unlike spatial pooling in 2D space, temporal pooling focuses on reducing the dimensionality of ${𝒱}_{t}$ along the temporal axis. The method selects the most salient features from all frames in the segment to produce a reduced, yet representative, feature representation.

In this work, the proposed temporal pooling method utilizes max pooling to extract the most salient features from the extracted feature matrix ${𝒱}_{t}$. This is achieved by taking the maximum values over a sequence of *n* frames, effectively filtering out less significant features and preserving the most significant spatiotemporal information. The proposed temporal max pooling is defined as follows:

(4) $$a_{t_{𝒦}}=\max\left({𝒱}_{t_{𝒦}}\right),\;𝒦=1,2,\ldots ,d$$

where $a_{t_{𝒦}}$ is the ${𝒦}^{th}$ feature at time step *t* over *n* frames. Temporal pooling is then applied repeatedly for all *d* features. Correspondingly, a matrix ${𝒱}_{t}$ is compressed as a fixed-length feature vector:

(5) $${\vec{a}}_{t}=\left(\begin{array}{c} a_{t_{1}}\\ a_{t_{2}}\\ \vdots \\ a_{t_{d}} \end{array}\right),\;{\vec{a}}_{t}\in R^{d}$$

By concatenating ${\vec{a}}_{t}$ over *T* segments, a matrix of features is obtained:

(6) $$𝒜=\left[\begin{array}{c} {\vec{a}}_{1},{\vec{a}}_{2},\ldots ,{\vec{a}}_{T} \end{array}\right],\;𝒜\in R^{d\times T}$$

where *𝒜* is the input of the proposed frameworks for classification purposes.

To improve the accuracy of human action recognition, the pre-trained Inception-ResNet-V2 model is fine-tuned on a human action video dataset. The fine-tuning process allowed us to adapt the model to better understand human actions and the dynamics of human movements. The human action videos are divided into *T* segments and the pre-trained Inception-ResNet-V2 model is expanded into *T* segments as well. The trainable parameters in the expanded model are shared across all segments, allowing us to capture the long-term temporal structure of human actions in a video. Next, fine-tuned temporal dense sampling (FTDS) is utilized to extract features from each segment by feeding a single frame randomly selected from each segment into the expanded pre-trained Inception-ResNet-V2 model. The extracted features are then passed into a 1D ConvNet network for representation learning and classification.

### 3.3. 1D Convolutional Neural Network (1DConvNet)

In recent years, convolutional neural networks (ConvNets) have been widely used for various applications. Among these, a 1DConvNet has been found to be an efficient solution for human action recognition as it requires fewer computations than 2DConvNets and recurrent networks such as long-short-term-memory (LSTM) networks. Therefore, in this work, we have proposed the use of a 1DConvNet as shown in Figure 1. In the proposed 1DConvNet, each ${\vec{a}}_{t}$ in the feature matrix *A* from the fine-tuned temporal dense sampling (FTDS) is treated as a separate channel. The filters in the 1DConvNet are trained adaptively through the convolution operation across time steps to extract meaningful spatiotemporal attributes from each channel. This results in the input sequence being projected into a feature space that is better suited for classification. Furthermore, the 1DConvNet has a significantly reduced number of trainable parameters compared to LSTM-based networks with similar experimental setups, making it a more computationally efficient option for human action recognition.

In 1DConvNet, the hidden states (${ℋ}^{l}$) associated with an $l^{th}$ hidden layer are formulated as below:

(7) $${ℋ}^{l}=\left\{\begin{array}{cc} {𝒲}_{H}^{l}*𝒜+b_{H}^{l}\;, & \mathrm{if}\;l=1\\ {𝒲}_{H}^{l}*{ℋ}^{l-1}+b_{H}^{l}\;, & l=2,3,\ldots ,L \end{array}\right.$$

where ∗ denotes the 1D convolution operation. ${𝒲}_{H}^{l}$ is the filter with the dimensionality of $f^{l}\times c^{l-1}\times c^{l}$, and biases are represented by ${\vec{b}}_{H}^{l}$. Here, $f^{l}$ and $c^{l}$ indicate the filter size and the number of filters in the $l^{th}$ hidden layer, respectively. As a result of the convolution operation, ${ℋ}^{l}$ is a matrix of hidden states in the $l^{th}$ layer with the dimensionality of $c^{l}\times {ℒ}^{l}$, where ${ℒ}^{l}$ is known as the length of a filter. In the case of l=1, the number of filters and the length of a filter are equivalent to *d* and *T*, respectively. Thereafter, the resultant hidden states are passed into the ReLU activation function:

(8) $${ℋ}^{l}=\left\{\begin{array}{cc} 0, & \mathrm{if}\;{ℋ}^{l}\leq 0\\ {ℋ}^{l}, & otherwise \end{array}\right.$$

Subsequently, the hidden states matrix at the last hidden layer ${ℋ}^{L}$ is flattened into a vector ${\vec{h}}^{L}$ to fully connect it with the output layer:

(9) $$\vec{z}={𝒲}_{z}\cdot {\vec{h}}^{L}+{\vec{b}}_{z},\;\vec{z}\in R^{M}$$

where $\vec{z}$ is the affine transformation of ${\vec{h}}^{L}$ with the associated weights matrix ${𝒲}_{z}$ and biases vector ${\vec{b}}_{z}$. Without a time axis, the predicted probability for a class is defined with a softmax function:

(10) $$p\left(\hat{y_{v}}|𝒜;𝒲,\vec{b}\right)=\frac{e^{z_{v}}}{\sum_{m=1}^{M}e^{z_{m}}}\;,v=1,2,\ldots ,M$$

By computing the probability for each class, the predicted probability distribution is defined as:

(11) $$\vec{\hat{y}}=\left(\begin{array}{c} p(\hat{y_{1}}|𝒜;𝒲,\vec{b})\\ p(\hat{y_{2}}|𝒜;𝒲,\vec{b})\\ \vdots \\ p(\hat{y_{M}}|𝒜;𝒲,\vec{b}) \end{array}\right)$$

where $\vec{\hat{y}}$ is a vector of predicted probability for each class. Following that, the classification of a human action video is predicted as the index of a class with the maximum estimated probability:

(12) $$\rho =\arg\max\vec{\hat{y}}$$

The backpropagation in the training process aims to minimize the cost function, i.e., negative log likelihood. To speed up and stabilize the training, it is optimized with mini-batch gradient descent using the Adam optimizer [39]. Formally, the update of weights and biases is defined as:

(13) $$\begin{array}{c} \theta \leftarrow \theta -\frac{\alpha }{\sqrt{{\hat{v}}_{t}}+\epsilon }*{\hat{m}}_{t} \end{array}$$

where θ is the parameter vector of weights and biases, i.e., *W* and $\vec{b}$. On the other hand, α is the initial learning rate, and ϵ is a small constant to avoid division by zero. For ${\hat{m}}_{t}$ and ${\hat{v}}_{t}$, they are defined to take into account the past gradients, to update the learning rate for each parameter accordingly:

(14) $$\begin{array}{c} m_{\tilde{t}}=B_{1}*m_{\tilde{t}-1}+\left(1-B_{1}\right)*\nabla \theta \\ v_{\tilde{t}}=B_{2}*v_{\tilde{t}-1}+\left(1-B_{2}\right)*{\left(\nabla \theta \right)}^{2} \end{array}$$

where ∇θ represents the gradients of the cost function with respect to the parameters. The moving averages of the gradients and squared gradients are denoted by $m_{\tilde{t}}$ and $v_{\tilde{t}}$, respectively. The default hyperparameter settings in the Adam optimizer are used as suggested in [39], where $B_{1}=0.90$, $B_{2}=0.999$, and $\epsilon =10^{-8}$. However, $m_{\tilde{t}}$ and $v_{\tilde{t}}$ tend to be very small with these hyperparameter settings during early iterations. For this reason, bias correction is introduced as:

(15) $$\begin{array}{c} {\hat{m}}_{\tilde{t}}=\frac{m_{\tilde{t}}}{1-{\left(B_{1}\right)}^{\tilde{t}}}\\ {\hat{v}}_{\tilde{t}}=\frac{v_{\tilde{t}}}{1-{\left(B_{2}\right)}^{\tilde{t}}} \end{array}$$

where $m_{\tilde{t}}$ and $v_{\tilde{t}}$ are scaled up during early iterations. As the training progresses, the effects of bias correction become trivial, thus $m_{\tilde{t}}$ and $v_{\tilde{t}}$ remain the same.

In order to mitigate overfitting in the proposed FTDS-1DConvNet, two regularization techniques are employed, namely L2 weight decay and max-norm constraint. The former involves decreasing the weights by a decay factor $\lambda_{1}$ when computing the gradients of the cost function with respect to the weights, while the latter involves scaling the weights with a certain ratio to ensure that the sum of their L2-norm does not exceed a threshold $\lambda_{2}$ after each iteration. These techniques play an important role in preventing the network from memorizing the training data, leading to a more generalized and robust model. The proposed FTDS-1DConvNet is outlined in Algorithm 1. A similar approach was presented by Banjarey et al. [40], where a 1D convolutional neural network was proposed for human action recognition. However, they simply passed the raw data into the network for learning, without any temporal segmentation or pooling. In contrast, our proposed FTDS-1DConvNet incorporates temporal segmentation and dense pooling to divide the video into segments and encode prominent spatial and temporal information into a fixed-length representation through max pooling along the temporal axis. The resulting output is then fed into a 1DConvNet for further representation learning and classification.
**Algorithm** **1** Training in the proposed FTDS-1DConvNet Framework1:Initialization: All parameters and hyperparameters.2:Fine-tuning: Pre-trained Inception-ResNet-V23:**for** epoch=1 to epoch=MaximumEpoch **do**4:      **for** iter=1 to iter=MaximumIteration **do**5:            𝒜← FTDS (refer to (6))6:            **Hidden states calculation:** ${ℋ}^{l}$ (refer to (8))7:            **Output layer calculation:** $\vec{z}$ (refer to (9))8:            **Probability calculation:** $\vec{\hat{y}}$ (refer to (11))9:            **Concatenation of the parameters:** $\theta =(W,\vec{b})$10:          **Gradients calculation:** $\frac{\alpha }{\sqrt{{\hat{v}}_{t}}+\epsilon }*{\hat{m}}_{t}$ (refer to (14) and (15))11:          **Parameters update:** $\theta \leftarrow \theta -\frac{\alpha }{\sqrt{{\hat{v}}_{t}}+\epsilon }*{\hat{m}}_{t}$12:    **end for**13:**end for**

## 4. Experiments and Analysis

This section provides an overview of the datasets, experimental setup, comparison with state-of-the-art methods, and visualization results used in the experiments.

### 4.1. Datasets

The UCF101 [41] and HMDB51 [42] datasets are widely used for evaluating the performance of human action recognition models. These datasets provide a diverse range of human actions, collected from real-world scenarios, which makes them ideal for testing the robustness of recognition models.

The UCF101 dataset contains 13,320 videos across 101 action classes, organized into 25 groups based on similarities in background and viewpoint. The HMDB51 dataset contains 6848 videos across 51 action categories, primarily collected from movies and other public sources such as YouTube. Both datasets categorize the human actions into several main types, including human–object interaction, body-motion, human–human interaction, playing musical instruments, and sports for UCF101, and facial actions, body movements, and object interactions for HMDB51.

### 4.2. Experimental Settings

To ensure fair comparison between experimental results, the same evaluation protocol, i.e., 3-split cross validation, is employed on both the UCF101 and HMDB51 datasets. This protocol has been recommended by the dataset providers in their respective publications [41,42]. In the 3-split cross validation protocol, the entire dataset is divided into three separate training and testing splits. In each iteration, two of the splits are combined to form the training set, while the remaining split is used as the testing set. The training and testing procedure is repeated three times, with a different split used as the testing set each time. The final performance evaluation is then reported as the average classification accuracy over the three splits:

(16) $$CA_{i}=\frac{\mathrm{Number}\;\mathrm{of}\;\mathrm{correctly}\;\mathrm{classified}\;\mathrm{testing}\;\mathrm{samples}\;\mathrm{in}\;i^{th}\;\mathrm{split}}{\mathrm{Total}\;\mathrm{number}\;\mathrm{of}\;\mathrm{testing}\;\mathrm{samples}\;\mathrm{in}\;i^{th}\;\mathrm{split}}\times 100\%$$

(17) $$Average\;CA=\frac{1}{3}\times \sum_{i=1}^{3}CA_{i}$$

where CA denotes the classification accuracy.

The optimal values of the hyperparameters of the proposed FTDS-1DConvNet are determined via experiments. The initial weight values are randomly chosen within the range of [−0.1,0.1] using a uniform distribution. The learning rate α is set to 0.0001 and the maximum number of training epochs is fixed at 100. Additionally, the number of segments *T* is set to 3.

In the experiments on the UCF101 dataset, it is observed that the performance of the FTDS-1DConvNet decreases with increasing number of filters $c^{l}$. The best results are achieved with a mini-batch size of K=32, one hidden layer (L=1), and 128 hidden states ($c^{1}=128$). On the other hand, for the HMDB51 dataset, the optimal average classification accuracy is reported with K=64, L=1, and $c^{1}=512$. To mitigate overfitting, the model is regularized using L2 weight decay with a decay factor $\lambda_{1}$ of 0.00004, and max-norm constraint with a threshold $\lambda_{2}$ of 0.4.

### 4.3. Experimental Results

In this study, various state-of-the-art methods based on ConvNet and LSTM have been compared with the proposed FTDS-1DConvNet. The evaluation protocol used was the average classification accuracy on 3-split cross validation, ensuring a fair comparison among the methods. To maintain consistency, all of the single-stream methods used only the RGB stream as their input.

The results of the experiments are presented in Table 1. While some methods have improved performance by using a two-stream input with spatial and temporal streams, the computation of optical flow for the temporal stream has been found to be computationally expensive. Despite achieving higher average classification accuracy, SVT [43] utilized a more complex vision transformer with a higher number of trainable parameters (86 M) as the backbone. In contrast, the proposed FTDS-1DConvNet achieved a promising average classification accuracy of 88.43% on the UCF101 dataset and 56.23% on the HMDB dataset, despite having a moderate number of parameters (56.23 M). This is a result of the FTDS-1DConvNet’s ability to effectively encode long-term spatiotemporal dependencies in human actions.

Among the compared existing works, shuttleNet achieved the highest performance [51]. This biologically inspired deep network proposed a new architecture that addresses the challenges of feedforward and feedback connections in traditional RNNs. Specifically, shuttleNet consists of several processors, each of which is a GRU associated with multiple groups of hidden states. Unlike traditional RNNs, all processors inside shuttleNet are loop connected, and an attention mechanism is employed to select the best information flow pathway. In contrast, our proposed FTDS-1DConvNet tackles the challenges of human action recognition through temporal segmentation and temporal dense sampling. The video is partitioned into segments and processed through a fine-tuned Inception-ResNet-V2 model, where max pooling is performed along the temporal axis to encode significant features into a fixed-length representation. This representation is then fed into a 1DConvNet for further representation learning and classification. Compared to RNN-based architectures, which use backpropagation through time (BPTT) for backpropagation, the FTDS-1DConvNet has a more stable backpropagation mechanism that is less susceptible to the vanishing gradients issue.

### 4.4. Visualization of the Proposed FTDS-1DConvNet

**Figure 2:**
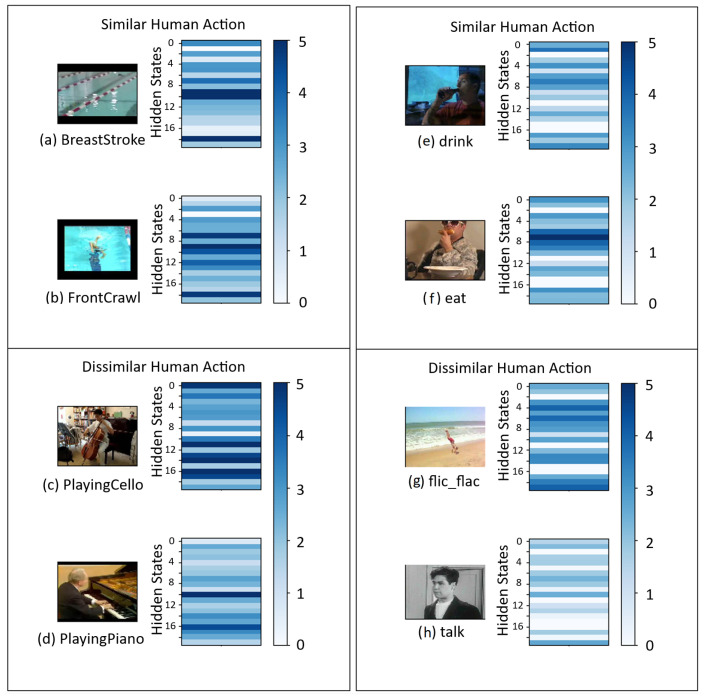
The heatmaps of the first 20 hidden states in FTDS-1DConvNet.

To better understand the workings of the proposed FTDS-1DConvNet, the hidden states have been visualized using heatmaps and t-SNE plots. As presented in Figure 2, a pair of similar and a pair of dissimilar human actions were selected from both the UCF101 and HMDB datasets. The results show that the dynamics of the hidden states of similar human actions tend to have similar distributions, while the dynamics of dissimilar human actions tend to have distinct distributions. This ability of the FTDS-1DConvNet to differentiate between similar human actions and represent their underlying dynamics through distinct distributions contributes to its high accuracy in human action classification.

**Figure 3:**
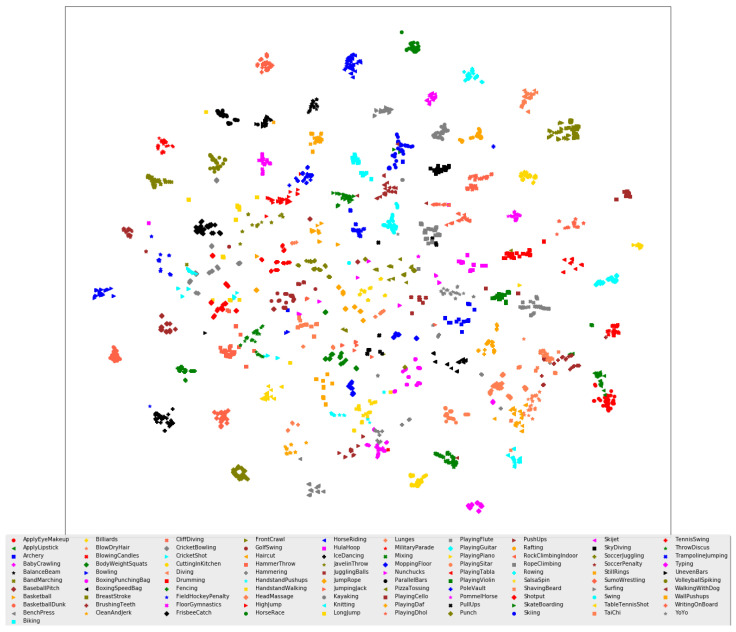
t-SNE and confusion matrix for the proposed FTDS-1DConvNet on the UCF101 dataset.

**Figure 4:**
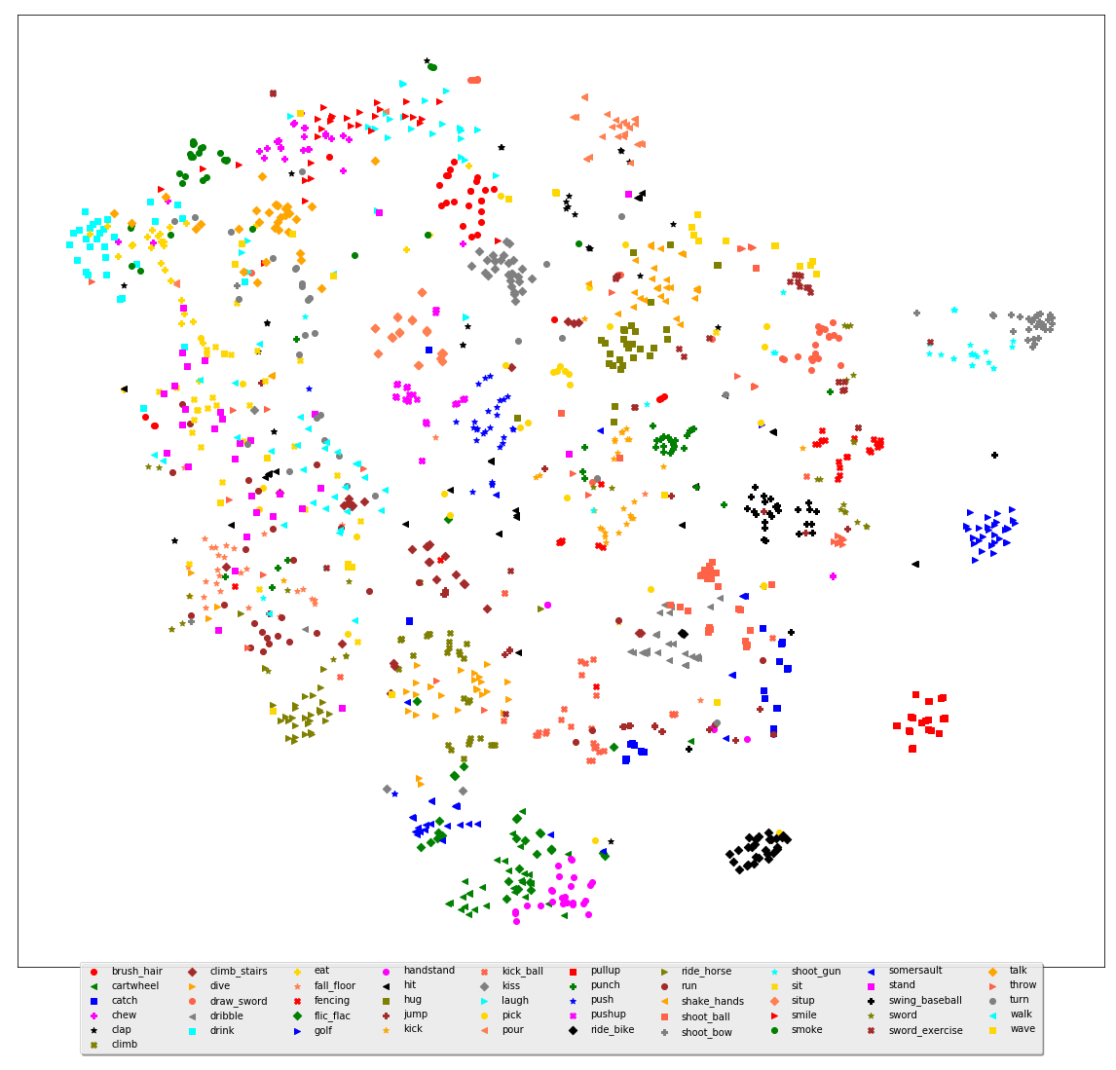
t-SNE and confusion matrix for the proposed FTDS-1DConvNet on the HMDB51 dataset.

The visualization of all hidden states with heatmaps is unfeasible due to the high number of hidden states in the proposed frameworks. To overcome this challenge, t-SNE is utilized to project the hidden states from all testing samples onto a two-dimensional space for better visualization. As shown in Figure 3 and Figure 4, clear clusters are formed for distinguishable human actions, and they exhibit linear separability, enabling easy classification by a softmax classifier in the final layer of the 1D ConvNet network. This indicates that the representation learning capability of neural-network-based architectures can convert initially non-linearly separable features into linearly separable ones. On the other hand, several clusters are grouped with other classes of human actions where the decision boundary between them is not linearly separable, such as *HammerThrow* and *ThrowDiscus* in the UCF101 dataset and *run* and *walk* in the HMDB51 dataset.

The proposed FTDS-1DConvNet transforms challenging human actions, such as *FrontCrawl*, *BreastStroke*, *HandstandPushups*, and *HandstandWalking* in the UCF101 dataset, into linearly separable ones. The representation learning in the proposed FTDS-1DConvNet reduces the variance of hidden states for human actions in the HMDB51 dataset such as *Throw*, *Talk*, and *Stand*, bringing the distributions of these samples closer together. This improvement in representation leads to increased classification accuracy for these human actions. The observations suggest that the gradient-based learning in the 1D ConvNet can compete with RNN models in encoding the spatiotemporal dependency of human actions.

## 5. Conclusions

In this paper, a novel approach for human action recognition, referred to as fine-tuned temporal dense sampling with 1D convolutional neural network (FTDS-1DConvNet), is proposed. The proposed FTDS-1DConvNet consists of two main stages: temporal segmentation and temporal dense pooling. The temporal segmentation step divides the video into a fixed number of segments. Each segment is then passed through a fine-tuned Inception-ResNet-V2 network for temporal dense sampling. During the temporal dense sampling, a max pooling operation is performed along the temporal axis within each segment. This process results in the prominent spatial and temporal information being encoded in a fixed-length representation. The output from the fined-tuned temporal dense sampling is then passed through a 1DConvNet for further representation learning and classification. By doing so, it enables the network to capture more complex and higher-level features in the temporal dimension. The effectiveness of the proposed FTDS-1DConvNet is demonstrated on two challenging datasets, UCF101 and HMDB51. The empirical results show that the proposed FTDS-1DConvNet outperforms other existing methods, such as the multi-stream ConvNet, 2DConvNet, and LSTM methods. This highlights the superiority of the proposed approach in capturing the spatiotemporal dependencies of human actions and projecting them into a more linearly separable space for better recognition and classification. The proposed FTDS-1DConvNet presents a promising direction for future research in the field of human action recognition, as it effectively integrates temporal segmentation and dense sampling with a 1DConvNet architecture to improve the accuracy of action recognition.

## Figures and Tables

**Table 1:** Comparison between average CA (%) and number of trainable parameters for the proposed FTDS-1DConvNet with other existing works using RGB stream.

Network(s)	No. of Trainable Parameters	UCF101	HMDB51
Multi-resolution ConvNet [25]	62 M	65.40%	-
Two-stream ConvNet [44]	17 M	73.00%	40.50%
Very Deep Two-streams ConvNet [30]	138 M	78.40%	-
C3D [45]	17.5 M	85.20%	-
Two-stream SR-ConvNet [33]	138 M	78.32%	-
Actions Transformation [46]	276 M	80.80%	44.10%
TSN [20]	11.29 M	85.10%	51.00%
ActionVLAD [47]	138 M	-	49.80%
LTC [37]	56 M	82.40%	-
Visual Attention [48]	14.31 M	-	41.31%
Unsupervised LSTM [49]	117 M	75.80%	44.10%
Long-term Recurrent ConvNet [16]	80.87 M	68.20%	-
Conv ALSTM [50]	222 M	79.60%	43.30%
Long-term LSTM [17]	15 M	82.60%	-
shuttleNet [51]	66.32 M	87.30%	54.20%
DPC [52]	32.6 M	75.70%	35.70%
Spatio-Temp [53]	58.3 M	61.20%	33.40%
VCOP [54]	58.3 M	65.60%	28.40%
RTT [55]	58.3 M	69.90%	39.60%
VideoMoCo [56]	14.4 M	78.70%	49.20%
SVT [43]	86 M	90.80%	57.80%
FTDS-1DConvNet (Ours)	56.23 M	88.43%	56.23%
